# Modulation of the activity of histone lysine methyltransferases and demethylases by curcumin analog in leukaemia cells

**DOI:** 10.1111/jcmm.17589

**Published:** 2022-10-27

**Authors:** Suhila Sawesi, Sridhar A. Malkaram, Zakaria Y. Abd Elmageed, Tamer E. Fandy

**Affiliations:** ^1^ Department of Pharmaceutical & Administrative Sciences, School of Pharmacy University of Charleston Charleston West Virginia USA; ^2^ Department of Mathematics & Computer Science West Virginia State University Institute West Virginia USA; ^3^ Department of Biomedical Sciences Edward Via College of Osteopathic Medicine (VCOM) Monroe Louisiana USA; ^4^ Present address: Health Informatics and Bioinformatics Graduate Program, School of Computing Grand Valley State University Allendale Michigan USA

**Keywords:** histone acetylation, histone methylation, histone methyltransferase, histone lysine demethylases, leukaemia

## Abstract

Curcumin is a known epigenetic modifier that demonstrated antitumor effect in different types of cancer. The poor solubility and metabolic stability are major drawbacks that limit its development as an antitumor agent. Dimethoxycurcumin (DMC) is a more soluble and stable curcumin analog. In this study, we compared the effect of both drugs on a variety of histone posttranslational modifications and on the activity of histone lysine methyltransferase (HKMTs) and demethylase (HKDMTs) enzymes that target the H3K4, H3K9 and H3K27 epigenetic marks. Mass spectrometry was used to quantitate the changes in 95 histone posttranslational modifications induced by curcumin or DMC. The effect of both drugs on the enzymatic activity of HKMTs and HKDMs was measured using an antibody‐based assay. Mass spectrometry analysis showed that curcumin and DMC modulated several histone modifications. Histone changes were not limited to lysine methylation and acetylation but included arginine and glutamine methylation. Only few histone modifications were similarly changed by both drugs. On the contrary, the effect of both drugs on the activity of HKMTs and HKDMs was very similar. Curcumin and DMC inhibited the HKMTs enzymes that target the H3K4, H3K9 and H3K27 marks and increased the activity of the HKDMs enzymes LSD1, JARID and JMJD2. In conclusion, we identified novel enzymatic targets for both curcumin and DMC that support their use and development as epigenetic modifiers in cancer treatment. The multiple targets modulated by both drugs could provide a therapeutic advantage by overcoming drug resistance development.

## 1 INTRODUCTION

Cancer is the leading cause of morbidity and mortality worldwide, contributing to more than 23 million new cases in 2019 and nearly 10 million deaths in 2022.^1^, ^2^, ^3^ Cancer is a multifaceted and diverse disease characterized by abnormal and uncontrolled cell proliferation. Cancer development is traditionally considered a result of genetic dysregulations. Nevertheless, epigenetic aberrations are now known to cooperate with genetic alterations to generate the cancer phenotype. Epigenetic modifications occur at a very early stage in neoplastic development and have a similar effect on stimulating malignant transformation and subsequent tumour growth as genetic mutations.^4^ Epigenetic changes comprise DNA methylation, histone modifications, chromatin remodellers, microRNAs and other components of chromatin that lead to gene silencing and/or activation.^5^ Epigenetic dysregulations, unlike genetic mutations, can be reversed and restored to their normal state. Such a reversible mechanism provides promising therapeutic targets to induce differentiation of tumour cells and/or complement the cytotoxicity of chemotherapy treatment.

A growing body of evidence proves that nutrients play a significant role in altering epigenetic modifications.^6^ Curcumin, a natural compound derived from the rhizome of the herb Curcuma longa (turmeric), is one of the most commonly used spices, remedies and colouring agents in the Asian continent and the Middle East. Curcumin demonstrated desirable therapeutic efficiency in many diseases, including cancer.^7^, ^8^ Despite curcumin's proven efficacy and safety against numerous experimental models at high concentrations, its poor bioavailability due to poor absorption, rapid metabolism and rapid systemic elimination has been shown to limit its therapeutic efficacy. Therefore, several curcumin analogs were synthesized to improve its bioavailability and stability.

Dimethoxycurcumin (DMC) is a more metabolically stable and potent analog of curcumin.^7^ Like curcumin, DMC was investigated for its cancer chemopreventive and chemotherapeutic properties.^8^, ^9^ The pharmacological activity of both curcumin and DMC could be attributed to their abilities to reverse the epigenetic alterations associated with the pathogenesis. Several studies documented the epigenetic alterations associated with the use of these compounds in leukaemia cells, such as their impact on DNA methylation,^9^, ^10^, ^11^, ^12^ histone acetylation^13^, ^14^ and miRNA modulation.^15^, ^16^, ^17^ However, their effect on histone methylation is not clear yet. In the present study, we compared the effect of both drugs on the enzymatic activity of the histone lysine methyltransferases (HKMTs) and the histone lysine demethylases (HKDMs) in leukaemia cells. The effect of both drugs was very similar and resulted in a decrease in HKMTs activity with a simultaneous increase in HKDMs activity at the H3K4, H3K9 and H3K27 epigenetic marks. These results highlight the potential of DMC as a promising epigenetic modifier and antitumor candidate and justify further studies to promote its progress to the clinical phases of drug development.

## 2 MATERIALS AND METHODS

### 2.1 Chemicals

Curcumin analytical standard grade (Sigma‐Aldrich, WI, USA) and DMC (Cayman, MI) were both dissolved in DMSO as a 10 mM stock solution, then aliquoted and stored in the dark at −80°C.

### 2.2 Cell culture and treatments

Three human leukaemia cell lines, human promyeloid leukaemia (HL60), Kasumi‐1 and human monocytic leukaemia (U937), were obtained from the American Type Culture Collection (ATCC, Manassas, VA) and cultured in RPMI‐1640 medium containing 10% foetal bovine serum (FBS) and 2.5 mM L‐glutamine. Cells were maintained in a humidified incubator with 5% CO_2_ at 37°C. Cells were treated with different micromolar concentrations of DMC (5, 2, 1, 0.5 and 0.2) or Curcumin (20, 10, 5, 2 and 1) or DMSO (control) for 48 hours followed by either apoptosis quantification or nuclear protein extraction for measuring HKMTs and HKDMs enzymatic activity.

### 2.3 Mass spectrometry

Samples were analysed on a triple quadrupole (QqQ) mass spectrometer directly coupled with an UltiMate 3000 Dionex nano‐liquid chromatography system as described previously.^18^ Targeted analysis of unmodified and modified histone peptides was performed three separate times for each sample. Raw data for replicates were transformed into z‐scores for each posttranslational modification (PTM) to generate the heatmap followed by cluster analysis using Pearson's correlation coefficient as distance measure.

### 2.4 Apoptosis quantitation

The percentage of live, apoptotic and necrotic cells was assessed by Flow cytometry using the Guava Nexin Reagent (EMD Millipore, MA) that contains 7‐aminoactinomycin D (7‐AAD) and Annexin‐V‐PE and analysed by Guava Nexin software as previously reported.^19^

### 2.5 Nuclear protein extraction

Nuclear extracts from treated and untreated HL60 and U937 leukaemia cells were prepared using the EpiQuikTM nuclear extraction kit (Epigentek, NY) as per the manufacturer's protocol. Approximately 2 × 10^6^ cells were collected and centrifuged at 1000 rpm for 5 min. The nuclear extract was prepared and stored at −80°C until used. The protein concentration of the nuclear extract was quantitated using the Quick Start Bradford Protein Assay (BioRad, Hercules, CA).

### 2.6 Enzymatic activity assay

HKMTs and HKDMs activities were quantitated by either fluorometric or colorimetric method that utilizes antibody‐based method and detects the different degrees of methylation of H3K4, H3K9 and H3K27 (Epigentek, Farmingdale, NY). The activity for the HKDM enzymes was determined fluorometrically using this formula:

$$\text{Activity}\;\left(\mathrm{OD}/\min/\mathrm{mg}\right)=\;\frac{\left(\text{Sample}\;\mathrm{RFU}-\text{blank}\;\mathrm{RFU}\right)}{\left(\text{protein amount}\;\left(\mathrm{ug}\right)x\;\min**\right)}\times 1000$$

**Incubation time = 120 minutes.

The activity of HMT enzymes was determined colorimetrically by the formula:

$$\text{Activity}\;\left(\mathrm{OD}/h/\mathrm{mg}\right)=\;\frac{\mathrm{OD}\;\left(\mathrm{no}\;\text{inhibitor}-\text{blank}\right)}{\left(\text{protein amount}\;\left(\mathrm{ug}\right)x\;\min**\right)}\times 1000$$

**Incubation time = 90 minutes, OD indicates optical density.

### 2.7 Statistical analyses

All cell culture experiments were performed in triplicates. Data represent the mean ± standard deviation (S.D.). Multiple comparisons between the effect of different concentrations of curcumin or DMC on the activity of HKMTs and HKDMs were performed using one‐way ANOVA followed by post hoc analysis using Bonferroni test.

## 3 RESULTS

### 3.1 Curcumin and DMC modulate histone methylation and acetylation in leukaemia cells

**FIGURE 1:**
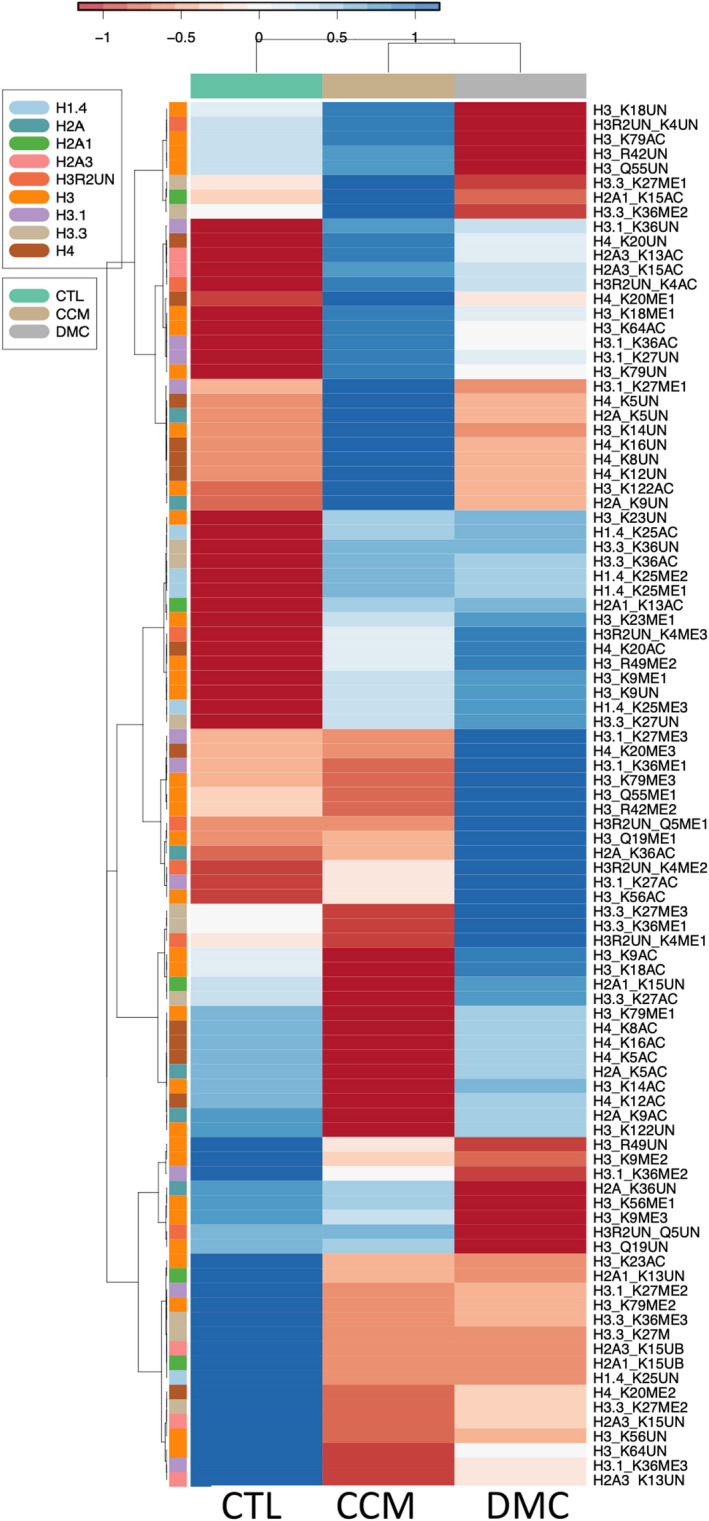
Cluster analysis of the changes in histone acetylation and methylation induced by curcumin and DMC. Kasumi‐1 leukaemia cells were treated with 10 μM curcumin or 1 μM DMC followed by bulk histone extraction and mass spectrometry analysis as described in methods. The data represent three separate mass spectrometry runs. The heatmap rows show the raw data of each histone modification for each replicate after transforming to z‐scores followed by cluster analysis as described under methods. CTL indicates the control sample, CCM indicates curcumin, and DMC indicates dimethoxycurcumin

Several studies reported the effect of curcumin on histone acetyltransferases (HAT) and histone deacetylases (HDACs). However, the effect of DMC on the enzymes that regulate histone methylation and acetylation is not clear. To address this, we studied the effect of both drugs on 95 different histone methylation and acetylation marks using mass spectrometry in leukaemia cells as described under methods. The heat map cluster analysis demonstrated that both drugs induced either similar or opposite effect on histone lysine methylation and acetylation (Figure 1). For instance, both drugs decreased H3.1 K27me2, H3.3 K36me3 and H3K79me2 methylation. On the contrary, curcumin increased H3.1 K27me1, H3K56me1, H3K9me3, H3.3 K27me1 and H3.3 K36me2, while DMC decreased all these histone marks. Additionally, both drugs increased H1.4 K25me1 and H1.4 K25me2 but to a different extent.

Beyond histone lysine methylation, histone methylation of arginine and glutamine residues was also modulated by both drugs (Figure 1), where curcumin increased the unmodified H3R42 and H3Q55 marks but DMC decreased both. On the contrary, curcumin decreased H3R42me2, H3Q55me1 and H3Q19me1, while DMC increased these marks. Similar effect of both drugs was observed in increased H3R49me2. The effect of DMC and curcumin on histone lysine acetylation was also not consistent with a similar effect.

Collectively, both drugs induced a variety of changes in histone lysine methylation and acetylation, in addition to changes in histone arginine and glutamine methylation.

### 3.2 Inhibition of H3K4 methylation by curcumin and DMC in leukaemia cells

**FIGURE 2:**
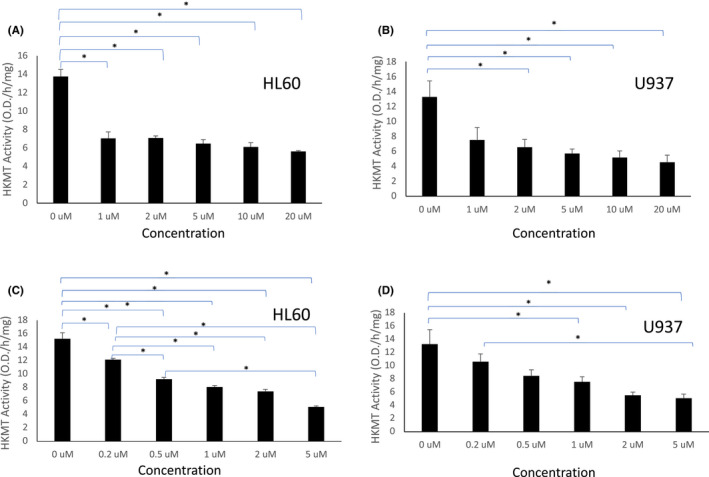
Curcumin and DMC inhibit the activity of HKMTs targeting H3K4. HL60 and U937 leukaemia cells were treated with different concentrations of curcumin (A and B, respectively) or DMC (C and D, respectively). The enzymatic activity of HKMTs targeting H3K4 was calculated as described under methods. Data represent the mean of three replicates ± SD. * and brackets indicate significant difference from the compared concentration at *p* < .05 using one‐way anova post hoc Bonferroni test

Histone lysine methyltransferases (HKMTs) regulate gene expression by inducing transcription repression or activation. H3K4 methylation is an epigenetic mark for euchromatin and transcription activation and is catalysed by enzymes like SET1, SET7/9, ALL‐1, MLL and SMYD3. Based on the mass spectrometry data above, we hypothesized that curcumin and DMC modulate the activity of the enzymes that methylate H3K4. To test our hypothesis, we measured the enzymatic activity of the HKMTs targeting the H3K4 epigenetic mark in two leukaemia cell lines in the presence and absence of curcumin and DMC as described under methods. Curcumin induced significant inhibition of HKMTs activity in both cell lines at the five different concentrations used (Figure 2A and B). Similarly, DMC induced significant inhibition of the activity of HKMTs targeting H3K4 in HL60 cells in all concentrations; however, inhibition was significant only in concentrations of 1 μM or higher in U937 cells (Figure 2C and D). To test the cytotoxicity of the concentrations used, we investigated apoptosis induction associated with using these concentrations. Curcumin induced minimal apoptosis in both cell lines at all concentrations used except for the highest concentration (20 μM), which induced significant apoptosis in both cell lines (Supplementary Figures S1A and B). On the contrary, DMC was more cytotoxic compared to curcumin and induced significant apoptosis at 5 μM concentration in both cell lines (Supplementary Figure S1C and D).

Taken together, both curcumin and DMC inhibited the activity of HKMTs targeting H3K4 in leukaemia cells and decreased H3K4 methylation.

### 3.3 Curcumin and DMC inhibit the methylation of the transcriptional repressor marks H3K9 and H3K27

**FIGURE 3:**
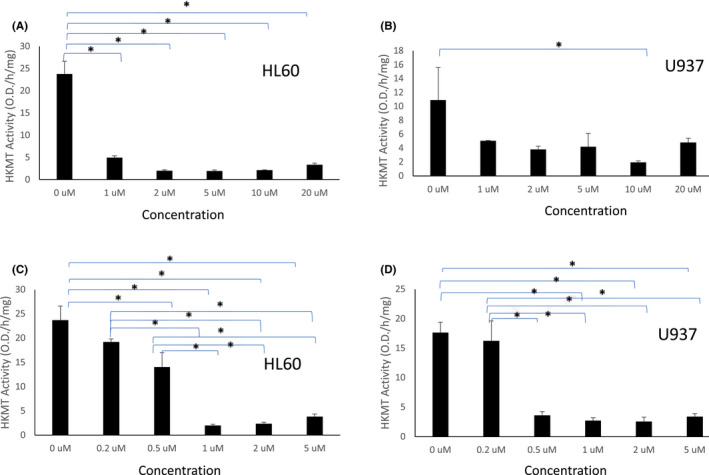
Curcumin and DMC inhibit the activity of HKMTs targeting H3K9. HL60 and U937 leukaemia cells were treated with different concentrations of curcumin (A and B, respectively) or DMC (C and D, respectively). The enzymatic activity of HKMTs targeting H3K9 was calculated as described under methods. Data represent the mean of three replicates ± SD. * and brackets indicate significant difference from the compared concentration at *p* < .05 using one‐way anova post hoc Bonferroni test

H3K9 methylation is associated with formation of heterochromatin and transcription repression. It is mediated by a group of HKMTs like G9a, SUV39‐h1, SUV39‐h2, SETDB1 and Dim‐5. Similarly, methylation of H3K27 induces transcriptional repression and is catalysed by HKMTs like G9a and EZH2. To investigate the effect of curcumin and its analog DMC on the methylation of both epigenetic marks, we measured the enzyme activity of the HKMTs that target both marks in two leukaemia cell lines, as described under methods. Both drugs inhibited the activity of the HKMTs that methylates H3K9. All the different concentrations of curcumin used significantly inhibited H3K9 methylation in HL60 cells (Figure 3A). On the contrary, U937 cells showed a similar effect at higher concentration of curcumin (10 μM) (Figure 3B). Similar to curcumin, DMC inhibited HKMT activity targeting H3K9 in both cell lines at all concentrations higher than 0.2 μM (Figure 3C and D).

**FIGURE 4:**
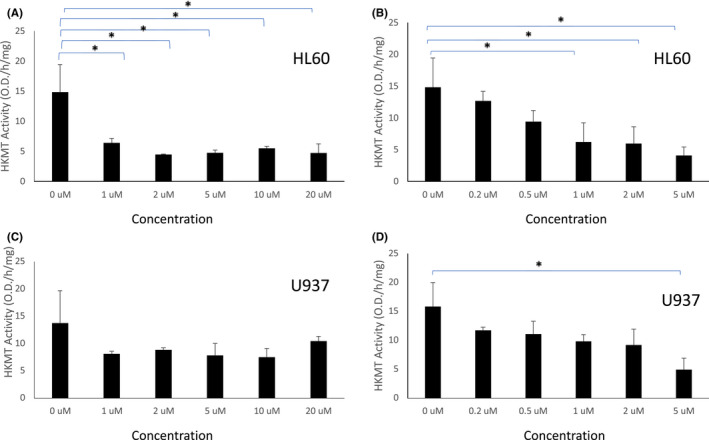
Curcumin and DMC inhibit the activity of HKMTs targeting H3K27. HL60 leukaemia cells were treated with different concentrations of curcumin or DMC (A and B, respectively) and U937 cells were treated with curcumin or DMC (C and D, respectively) followed by measuring the activity of the HKMTs that methylates H3K27. The enzymatic activity was calculated as described under methods. Data represent the mean of three replicates ± SD. * and brackets indicate significant difference from the compared concentration at *p* < .05 using one‐way anova post hoc Bonferroni test

Inhibition of H3K27 methylation was similarly observed in HL60 cells by the two drugs (Figure 4A and B), where DMC induced significant inhibition at 1 μM concentration or higher. On the contrary, the changes observed in U937 cells with both drugs were not statistically significant at *p* < .05 except at the highest concentration of DMC (Figure 4C and D). Taken together, both curcumin and DMC inhibited the methylation of both H3K9 and H3K27 in leukaemia cells.

### 3.4 Curcumin and DMC increase the activity of the histone lysine demethylase enzymes

**FIGURE 5:**
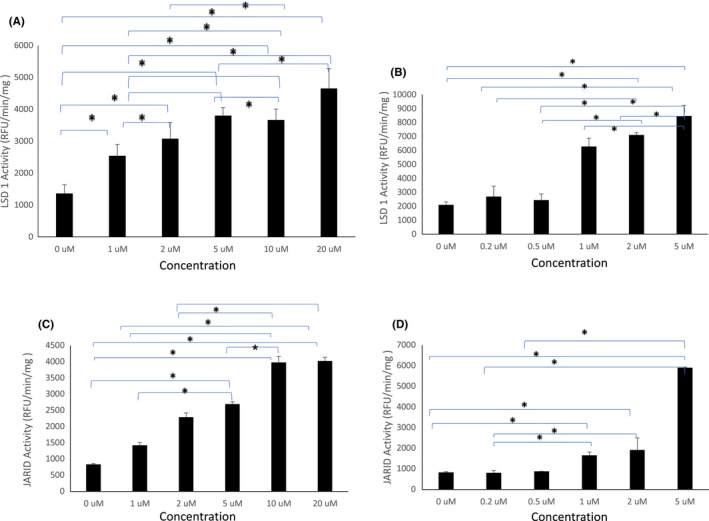
Curcumin and DMC increase the activity of the histone lysine demethylases LSD1 and JARID. HL60 leukaemia cells were treated with different concentrations of curcumin or DMC followed by measuring the activity of LSD1 (A and B, respectively) or JARID (C and D, respectively). The enzymatic activity was calculated as described under methods. Data represent the mean of three replicates ± SD. * and brackets indicate significant difference from the compared concentration at *p* < .05 using one‐way anova post hoc Bonferroni test

The methylation of histones is a dynamic process regulated by both HKMTs and HKDMs. LSD1 and JARID enzymes are histone lysine demethylases that target H3K4, where LSD1 targets the mono and dimethylated H3K4 and JARID1 targets trimethylated H3K4. On the contrary, H3K9 demethylation is catalysed by the JMJD1 and JMJD2 family of enzymes, where JMJD1 family (1A, 1B and 1C) removes di and mono‐methylation from H3K9 and JMJD2 family (2A – 2F) removes trimethylation from H3K9 and H3K36. Since we observed a decrease in HKMTs activity targeting the H3K4 and H3K9 marks by curcumin and DMC in leukaemia cells, we hypothesized that both drugs may also modulate the activity of HKDMs targeting H3K4 and H3K9. Accordingly, we examined the effect of curcumin and DMC on the activity of three demethylase enzymes (LSD1, JARID1 and JMJD2) that target the H3K4 and H3K9 marks. Curcumin and DMC increased the activity of LSD1 in HL60 cells (Figure 5A and B, respectively) and U937 cells (Supplementary Figure S2A and B). Similarly, JARID activity was increased by curcumin and DMC (at concentrations higher than 0.5 μM) in HL60 cells (Figure 5C and D, respectively) and in U937 cells (Supplementary Figure S2C and D). Furthermore, JMJD2 activity was increased by both drugs in both HL60 cells (Supplementary Figure S3A and B) and in U937 cells (Supplementary Figure S3C and D). In summary, both curcumin and DMC increase the activity of different demethylase enzymes targeting H3K4 and H3K9 marks and complement the simultaneous decrease in the activity of HKMTs targeting these marks.

## 4 DISCUSSION

Curcumin and its analogs demonstrated antitumor effect in different types of cancer. The poor solubility and bioavailability of curcumin stimulated the development of analogs with better metabolic profile and water solubility. DMC is a more metabolically stable curcumin analog that was shown to induce the re‐expression of promoter‐methylated genes as a single agent and in combination with the DNA methyltransferase (DNMT) inhibitor decitabine.^10^ To further explore the epigenetic changes induced by both drugs, we tested their effect on the activity of the epigenetic writers and erasers of histone lysine methylation in leukaemia cells. Both drugs significantly decreased the activity of different HKMTs with simultaneous increase in the activity of the histone demethylases LSD1, JARID and JMJD2. These changes will induce a decrease in methylation at the H3K4 and H3K9 marks with consequent changes in gene expression that could contribute to their antitumor effect.

DMC is a promising lipophilic curcumin derivative that showed better systemic bioavailability and similar antitumor activity.^20^ Unfortunately, most of the studies focused on the cytotoxicity^21^, ^22^ and the generation of reactive oxygen species^23^, ^24^ by DMC with only few studies that highlighted the epigenetic changes induced by DMC or curcumin.^9^, ^10^ For instance, curcumin induced the mRNA and protein level of JMJD3 in human neuroblastoma cells with consequent decrease in H3K27me3 at the BDNF promoter region and increase in BDNF expression, suggesting a protective role of curcumin in Alzheimer's disease.^25^ Additionally, curcumin reduced the expression of EZH2 in cell line and mouse models of myelodysplastic syndrome with consequent decrease in H3K27me3.^26^ Inhibition of expression of the arginine methyltransferase PRMT5 with simultaneous decrease in dimethylation of the H4R3 mark was also observed in lung and breast cancer cells after treatment with curcumin.^27^ None of the above studies measured the activity of the epigenetic enzymes and focused on measuring their expression. Our results are concordant with these previous reports, but we provided direct evidence for the changes in the enzymatic activity of the enzymes that target these epigenetic marks, which further emphasizes the potential of DMC as an epigenetic modifier with improved pharmacokinetics compared to curcumin.

HKMT inhibitors demonstrated antitumor effects in different types of cancer. Inhibition of the euchromatic histone lysine methyltransferases 1 and 2 (EHMT1/2) enhanced the antiproliferative activity of HDAC inhibitors in colorectal cancer and reduced H3K9 methylation.^28^ EZH2 is the enzymatic catalytic subunit of polycomb repressive complex 2 (PRC2) that catalyses the trimethylation of H3K27. Gain‐of‐function mutation in the catalytic SET domain of EZH2 was detected in lymphoma and increased H3K27me3.^29^ Additionally, EZH2 mutations were associated with poor overall survival in myeloid neoplasms.^30^ Inhibitors of EZH2 and dual inhibitors that can target its analog EZH1 demonstrated antitumor activity in both solid tumours and haematologic malignancies.^31^ Furthermore, inhibitors of lysine methyltransferases that target H3K4 like MLL, SMYD and SET7/9 demonstrated antitumor activity in leukaemia, breast cancer and prostate cancer.^32^ In this study, DMC and curcumin decreased the methylation of the three epigenetic marks H3K4, H3K9 and H3K27, which may contribute to their observed antitumor effect in different types of cancer. However, it is not clear if inhibiting the three epigenetic marks simultaneously could provide an advantage over the specific targeting of one of them.

The observed increase in histone lysine demethylases activity with the simultaneous decrease in HKMTs activity favours the decrease in methylation at the H3K4, H3K9 and H3K27 marks. However, the increase in the HKDMs activity could be an undesirable effect and against the antitumor effect of both drugs. In support of that, LSD1 is overexpressed in haematologic malignancies and its inhibition induced differentiation of AML cells through the downregulation of the chromatin protein GSE1.^33^ Similarly, the oncogenic role of JMJD2 in AML, hepatocellular, breast cancer and colorectal cancer was also reported.^34^, ^35^ Nonetheless, the demethylase activity of JMJD2 was shown to be essential for the long‐term maintenance of normal haematopoiesis.^36^ On the contrary, the impact of JARID1 activation on the antitumor effect of DMC and curcumin is not clear because of the oncogenic and tumour suppressive functions of JARID1 proteins, which are contingent on the protein isoform (JARID1A, JARID1B, JARID1C, JARID1D) and cell context.^37^

Although the effect of curcumin and DMC was very similar on the enzymatic activity of HKMTs and HKDMs, their effect on methylation and acetylation of different histone residues using mass spectrometry was heterogeneous. This can be attributed to the drawback of the mass spectrometry analysis method which captures the final state of the epigenetic mark without any information about the activity of the enzymes that methylate or demethylate the mark. On the contrary, the enzyme activity assay does not capture the final state of the epigenetic mark and only focuses on enzyme activity. Also, it is not clear if the mass spectrometry data are cell line specific or similar changes will be observed in other leukaemia cell lines.

In conclusion, we identified HKMTs and HKDMs as novel targets for curcumin and its analog DMC. The promiscuous targets of both drugs could be advantageous in treating cancer by overcoming drug resistance development and could also yield undesirable effects. Epigenetic modifiers like 5‐Azacytidine and decitabine are also highly non‐specific but demonstrated clinical efficacy in haematologic malignancies. Our data support further studies on DMC in animal models of AML and its potential to proceed into future clinical trials.

## AUTHOR CONTRIBUTIONS

**Suhila Sawesi:** Data curation (lead); formal analysis (lead); writing – original draft (supporting); writing – review and editing (supporting). **Sridhar A. Malkaram:** Data curation (lead); formal analysis (lead); writing – review and editing (supporting). **Zakaria Y. Abd Elmageed:** Data curation (equal); resources (equal); writing – review and editing (equal). **Tamer E. Fandy:** Conceptualization (lead); funding acquisition (lead); investigation (lead); methodology (lead); project administration (lead); supervision (equal); writing – original draft (equal); writing – review and editing (equal).

## CONFLICT OF INTEREST

The authors have no financial interest or benefit to disclose.

## Supporting information


**Figure S1** Curcumin and DMC are highly non‐cytotoxic to leukaemia cells. HL60 and U937 leukaemia cells were treated with different concentrations of curcumin (1a and 1b, respectively) and DMC (1c and 1d, respectively) for 48 h and apoptosis induction was measured as described under methods. Data represent the average of 3 replicates ± SD. * indicates significant difference from the control at *p* < .05
**FIGURE S2** Curcumin and DMC increase the activity of the histone lysine demethylases LSD1 and JARID. U937 leukaemia cells were treated with different concentrations of curcumin or DMC followed by measuring the activity of LSD1 (2a and 2b, respectively) or JARID (2c and 2d, respectively). The enzymatic activity was calculated as described under methods. Data represent the mean of three replicates ± SD. * and brackets indicate significant difference from the compared concentration at *p* < .05 using One‐way anova post hoc Bonferroni test
**FIGURE S3** Curcumin and DMC increase the activity of the histone lysine demethylases JMJD2. HL60 leukaemia cells were treated with different concentrations of curcumin and DMC (3a and 3b, respectively) followed by measuring the activity of JMJD2. Similarly, U937 cells were treated with different concentrations of curcumin and DMC (3c and 3d, respectively) followed by measuring the activity of JMJD2. The enzymatic activity was calculated as described under methods. Data represent the mean of three replicates ± SD. * and brackets indicate significant difference from the compared concentration at *p* < .05 using One‐way anova post hoc Bonferroni test

## Data Availability

The data that support the findings of this study are available from the corresponding author upon reasonable request.
