# Supplementary material for: Modulation of the activity of histone lysine methyltransferases and demethylases by curcumin analog in leukaemia cells
Source: J Cell Mol Med. 2022 Oct 27;26(22):5624–33. doi: 10.1111/jcmm.17589 (PMC9667515; doi:10.1111/jcmm.17589)

**Supplementary Data**

**Modulation of the activity of histone lysine methyltransferases and demethylases by curcumin analog**

Suhila Sawesi^1^, Sridhar A. Malkaram^2^, Zakaria Y. Abd Elmageed^3^, Tamer E. Fandy^1,*^

^1^Department of Pharmaceutical & Administrative Sciences, School of Pharmacy, University of Charleston, Charleston, WV. ^2^Department of Mathematics & Computer Science, West Virginia State University, Institute, WV. ^3^Edward Via College of Osteopathic Medicine (VCOM), Monroe, LA.

***Corresponding Author:**

Tamer E. Fandy, Ph.D., BCGP, FCP

Department of Pharmaceutical & Administrative Sciences

School of Pharmacy

University of Charleston

Charleston, WV 25304

Tel.: (304)357-449

E-mail: tamerfandy@ucwv.edu

**Figure 1. Curcumin and DMC are highly non cytotoxic to leukemia cells.** HL60 and U937 leukemia cells were treated with different concentrations of curcumin (1a and 1b, respectively) and DMC (1c and 1d, respectively) for 48 h and apoptosis induction was measured as described under methods. Data represent the average of 3 replicates ± SD. * indicates significant difference from the control at p < 0.05.

1a.

1b.

1d.

1c.

**Figure 2: Curcumin and DMC increase the activity of the histone lysine demethylases LSD1 and JARID.** U937 leukemia cells were treated with different concentrations of curcumin or DMC followed by measuring the activity of LSD1 (2a and 2b, respectively) or JARID (2c and 2d, respectively). The enzymatic activity was calculated as described under methods. Data represent the mean of three replicates ± SD. * and brackets indicate significant difference from the compared concentration at p < 0.05 using 0ne-way ANOVA post hoc Bonferroni test.


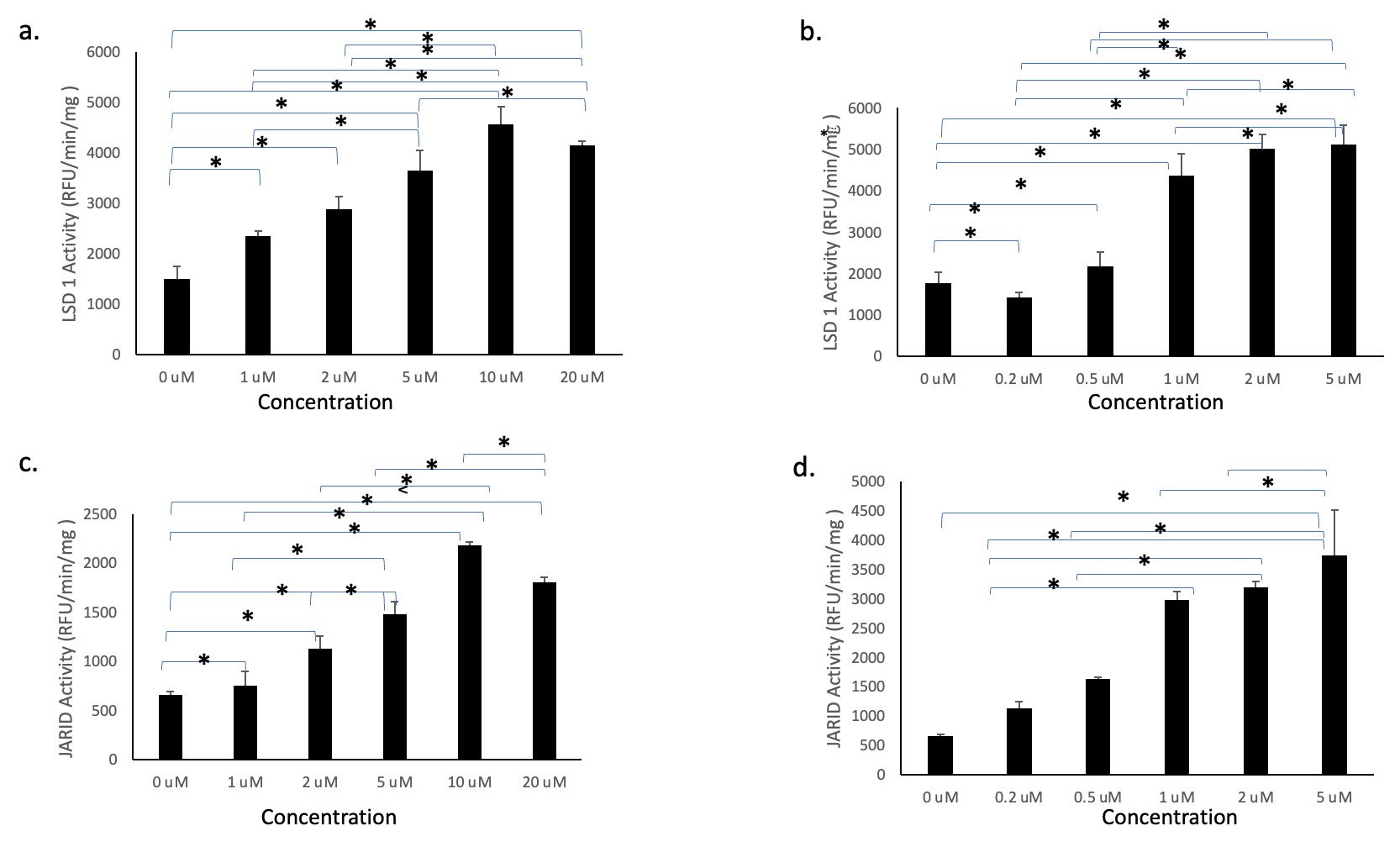


**Figure 3: Curcumin and DMC increase the activity of the histone lysine demethylases JMJD2.** HL60 leukemia cells were treated with different concentrations of curcumin and DMC (3a and 3b, respectively) followed by measuring the activity of JMJD2. Similarly, U937 cells were treated with different concentrations of curcumin and DMC (3c and 3d, respectively) followed by measuring the activity of JMJD2. The enzymatic activity was calculated as described under methods. Data represent the mean of three replicates ± SD. * and brackets indicate significant difference from the compared concentration at p < 0.05 using 0ne-way ANOVA post hoc Bonferroni test.


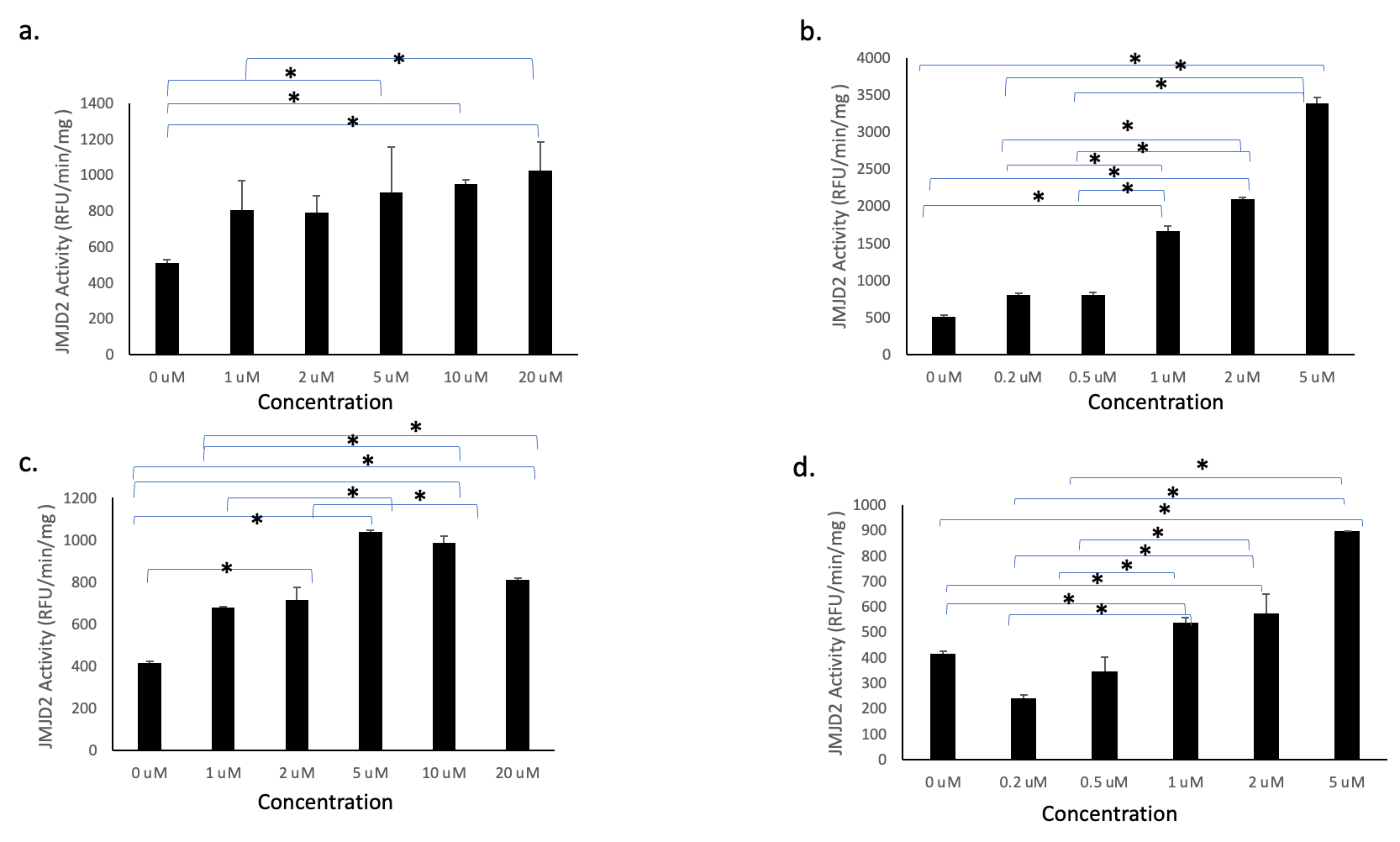

Supplement: Supplementary file 1 — Figure S1 Curcumin and DMC are highly non‐cytotoxic to leukaemia cells. HL60 and U937 leukaemia cells were treated with different concentrations of curcumin (1a and 1b, respectively) and DMC (1c and 1d, respectively) for 48 h and apoptosis induction was measured as described under methods. Data represent the average of 3 replicates ± SD. * indicates significant difference from the control at p < .05 FIGURE S2 Curcumin and DMC increase the activity of the histone lysine demethylases LSD1 and JARID. U937 leukaemia cells were treated with different concentrations of curcumin or DMC followed by measuring the activity of LSD1 (2a and 2b, respectively) or JARID (2c and 2d, respectively). The enzymatic activity was calculated as described under methods. Data represent the mean of three replicates ± SD. * and brackets indicate significant difference from the compared concentration at p < .05 using One‐way anova post hoc Bonferroni test FIGURE S3 Curcumin and DMC increase the activity of the histone lysine demethylases JMJD2. HL60 leukaemia cells were treated with different concentrations of curcumin and DMC (3a and 3b, respectively) followed by measuring the activity of JMJD2. Similarly, U937 cells were treated with different concentrations of curcumin and DMC (3c and 3d, respectively) followed by measuring the activity of JMJD2. The enzymatic activity was calculated as described under methods. Data represent the mean of three replicates ± SD. * and brackets indicate significant difference from the compared concentration at p < .05 using One‐way anova post hoc Bonferroni test [file JCMM-26-5624-s001.docx]
